# Infections in the Elderly Critically-Ill Patients

**DOI:** 10.3389/fmed.2019.00118

**Published:** 2019-06-06

**Authors:** Mert Esme, Arzu Topeli, Burcu Balam Yavuz, Murat Akova

**Affiliations:** ^1^Section of Geriatric Medicine, Department of Medicine, Hacettepe University School of Medicine, Ankara, Turkey; ^2^Section of Intensive Care, Department of Medicine, Hacettepe University School of Medicine, Ankara, Turkey; ^3^Department of Infectious Diseases and Clinical Microbiology, Hacettepe University School of Medicine, Ankara, Turkey

**Keywords:** elderly, severely ill, infection, antimicrobial stewardship, immunosenescence

## Abstract

Infections are leading causes of morbidity and mortality in the advanced aged. Various factors including immunosenescens, comorbid chronic diseases, and alterations in normal physiological organ functions may modify the frequency and severity of infections in elderly patients. Normal body reactions to ensuing infection, such as increased body temperature, may be blunted in those patients causing difficulties in differential diagnosis between infection and other diseases. In severe infections the respiratory and urinary tracts are the most frequently involved systems which may be accompanied by severe sepsis. Bacteremia and sepsis are also associated with indwelling vascular catheters in the elderly who are admitted to the intensive care unit (ICU). Older patients are more vulnerable to the *Clostridioides difficile* infection, as well. Although the general management of infections in severely ill elderly patients is not different than in younger patients, meticulous care in fluid management and careful individualized optimization in antibiotic therapy, along with the other principals of antimicrobial stewardship are warranted in order to prevent increased mortality caused by infection. Organized team management when treating critically ill elderly patients in the ICU is essential and will reduce the morbidity and mortality due to infection in such patients.

## Introduction

Aging is associated with a variety of physiological changes and progressive decline in physiological homeostasis, both of which lead to alterations in organ functions, functional decline, multimorbidity, and frailty (1, 2). Age dependent changes in the immune system are referred to as immunosenescence and this may affect the organism's ability to overcome external stressors. Immunosenescence is present in all older adults in varying degrees and there is a link between the degree of frailty and immunocompetency (3). As the immune system ages and the normal capabilities of defense against infections, malignant or autoreactive cells declines, increased susceptibility to infections, malignancy, autoimmune disorders, and impaired wound repair follow (1, 4). Many older adults have mild degrees of immunosuppression as a result of immunosenescence, together with, age related organ changes, comorbidities, geriatric syndromes, frailty, malnutrition, functional dysfunction and, polypharmacy, all of which affect the prognosis of geriatric patients with infectious diseases (2, 3). Physiological age-related changes in the immune system predisposing infections are summarized in Table 1.

**Table 1:** Physiologic changes in the immune system that occur with aging (1–4).

	**Inappropriate elevation with age**	**Inappropriate decrease with age**
Innate immune system	NK cells: CD56^dim^ NK cell number	Anatomical and biochemical barriers: Regeneration, sweat production, and barrier function of skin and mucus
		Hematopoietic tissue: total number of hematopoietic stem cells (HSCs) in the bone marrow, total bone marrow hematopoietic tissue, the proliferative capacity of HSCs
		Macrophages: Bone marrow precursors, phagocytic capacity, and oxidative killing activity of macrophages
		Neutrophils: chemotactic responses, migration capacity, phagocytic capacity, and superoxide generation
		NK cells: CD56^bright^ NK cell number
Adaptive immune system		
T cells	Number of CD8 T cells	Involution of the thymus gland
		Number of thymic precursors
		Number of naïve T cells
		T cell repertoire
		Functional activity of Regulatory T cells (Treg)
		Number of CD4 T cells
		Number of CD28 T cells
B cells	Autoreactive serum antibodies	B cell precursors in the bone marrow
		Number of B cells
		Plasma cell differentiation
		Specific antibody production
		B cells' response to antigen exposure
		The diversity of the B cell repertoire
		Opsonizing capacity of immunglobulins

In the past decades, there has been an increase in the number of elderly patients admitted to the intensive care unit (ICU). Currently, the median age of critically-ill patients approaches 65 years and the proportion of the very old (≥80 years of age) among them is on rise. This situation poses ethical and practical challenges both before ICU admission and during ICU stay, increasing healthcare costs (5). Infections and related complications such as sepsis and acute respiratory failure are both observed at admission and are frequently observed during the ICU stay in this patient population.

Elderly critically ill patients are faced with increased morbidity and mortality even if they survive the intensive care episode, although, there is still an ongoing debate whether age or underlying physiologic reserve, frailty, etc., are actually the causes of poor outcomes. These patients have poorer hospital survival than patients younger than 65 years of age, when other confounding factors such as disease severity score, invasive procedures, and comorbidities are controlled (6).

## Predisposing factors and vulnerability for severe infections in elderly

Immunosenescence, comorbidity, malnutrition, and social determinants of health (e.g., nursing home residence, poor access to care) increase the risk of infections in older adults (2). Infection is the primary cause of death in one third of individuals aged 65 years and over. Infection has also a significant effect on morbidity in geriatric patients, exacerbates underlying diseases, and leads to functional decline. Many of the biological, cultural and, social factors indicate that older adults are susceptible to infection and have worse results when they are infected. These factors also alter the presentation of infectious syndromes in the elderly and may lead to treatment modifications (7).

Impaired immunity is much more associated with disease burden than the chronological age. Older adults with chronic diseases (e.g., diabetes mellitus, chronic obstructive pulmonary disease (COPD) or heart failure) are more susceptible to common infections and exhibit weaker vaccine response than those without underlying health problems (8). Age-related organ-specific physiologic changes also increase the risk of infection and affect the clinical presentation of infectious syndromes. Table 2 summarizes the characteristics of major infectious diseases including predisposing factors and preventive and therapeutic measures in the elderly as compared with younger patients.

**Table 2:** Characteristics of major infections in the elderly as compared with the younger patients (1, 2, 7, 9–17).

**Type of infection**	**Predisposing factors for specific infections in elderly**	**Major differences in elderly as compared with younger patients (12, 18–22)**	**Preventive and theraputic measures in elderly**
Respiratory infections	Decreased: • Cough and other protective reflexes • Lung elasticity • Mucociliary clearance • Ig levels in respiratory secretions Increased levels of circulating inflammatory cytokines • Malnutrition	• Increased risk and frequency of pneumonia in elderly (4–11 X higher than those of <65 years) • More severe disease and high mortality • Co-infections are more frequent in elderly admitted to ICU with influenza • More resistant bacteria as the causative agents of pneumonia in LTCF • More ICU admission in those >85 years • Mortality due to VAP is higher in those >65 years • Duration of ICU stay and MV are longer • Weaning from MV is more difficult • Poor vaccine response	• Annual influenza vaccination • Pneumococcal vaccination • Careful observation of drug interaction and toxicities • Drug dosage adjustments according to accompanying co-morbidities
Urinary tract infections	Mechanical changes: • Reduced bladder capacity • Involuntary contractions • Decreased urine flow • Urinary stasis due to prostate hypertrophy or bladder prolapsus Uroepithelial changes	• High prevalence of bacteriuria • Increased bacterial adhesion to uroepithelium	Avoid unnecessary urinary catheterization Avoide treatment of asymptomatic bacteriuria
Skin and soft tissue infections	• Malnutrition, increased catabolism • Collagen and subcutaneous tissue loss • Decreased size of blood vessels in dermis • Decreased water binding capacity of stratum corneum • Decreased derma-epidermal adhesion • Decreased mobility and bedridden status	Slower wound healing Increased incidence of bed sores and pressure ulcers	Minimize prolonged pressure Keep skin dry and clean
Bacteremia and sepsis	• Immunosupression due to immunosenescence • Frequent use of iv lines in criticaly ill elderly	Increased bacteremia and sepsis	Avoid unucessary iv catheter insertion and prompt removal as indicated by the type of bacteremia or fungemia
*Clostridioides difficile* infection (CDI)	• Decreased gastric acidity and intestinal motility • Changes in intestinal microbiome (decrease in Bifidobacteria and increase in Enetrobacteriaceae) • Slow recovery of microbiome after antibiotic usage	• Higher mortality and morbidity • More recurrences • More severe disease	Avoid unnecessary and prolonged antibiotic use

## Epidemiological characteristics of frequently seen infections in the critically ill elderly

The age of patients significantly affects the type of infections and related microbial etiology in those who are older than 65. In a secondary analysis of data from an international, observational, point prevalence study (EPIC II) revealed that out of infected patients whom comprised 51.4% of the whole cohort, almost half (48.7%) were aged 65 and older (9). There was no difference in disease severity among age groups (18–44, 45–64, 65–74, 75–84, ≥85), however, ≥85 years of age was an independent risk factor for admission to the ICU and hospital mortality. It has been shown that patients who are older than 85 had fewer bloodstream and central nervous system infections, but more intraabdominal infections as compared to younger patients. More gram-negative bacteria were isolated in >85 year-olds and in-hospital mortality was much higher in this age group (9).

### Respiratory Infections

The rate of pneumonia increases rapidly with age. Bacteria are responsible for the majority of pneumonia in older adults. Although 1 out of every 1,000 cases needs to be hospitalized, this rate is 12 in 1,000 for patients over 75 years and 33 in 1,000 for nursing home patients. As in many diseases, the clinical manifestation of pneumonia may be atypical and subtle in older patients. Sore throat, headache following nose run, weakness, fever, chills, shortness of breath, chest pain, and productive cough are typical clinical symptoms for pneumonia. However, these symptoms may not be seen especially in the frail aged or in patients with cognitive impairment and multi-morbidity. Complaints may include fatigue, loss of appetite, anorexia, confusion, delirium, falls, or sometimes urinary incontinence. Older adults may not have fever even in severe infections (7).

Around 50% of all CAP occurs in adults older than 65 years of age (23). Severe sepsis may be present in approximately one-third of all patients with community acquired pneumonia (CAP) at the time of admission to the emergency room (18). A chest X-ray is necessary for diagnosing pneumonia and its complications, however a computed tomograpy may be needed to exclude an underlying malignancy and/or for precise diagnosis of alveolar infiltration (7). Although half of the patients remain without a microbiological diagnosis of CAP, *Streptococcus pneumoniae* is the leading causative agent. Chronic obstructive pulmonary disease predisposes pneumonia caused by *Haemophilus influenzae, Moraxella catarrhalis*, and *Legionella pneumophila*. Those residing in long-term care facilities are prone to be colonized by resistant bacteria due to their frequent exposure to broad spectrum antibiotics and underlying comorbidities which in turn cause severe pneumonic syndromes. *Chlamydophila pneumoniae* and viruses including influenza, rhinovirus, and human metapneumovirus can be detected frequently (18, 24). An increased rate of co-infections in the elderly with influenza admitted to the ICU has been reported and is associated with 28-day and hospital mortality (25). Polymicrobial pneumonia is also frequently encountered in elderly patients admitted to the ICU (26).

The European Union-Ventilator Associated Pneumonia (EU-VAP) study conducted in 27 European ICUs revealed that prevalence of VAP did not differ between age groups (middle age: 45–64, old age: 65–74, very old age: ≥75) and age was not an independent risk factor for VAP (27). However, mortality was higher in old and very old patients compared to younger patients (51 vs. 35%, *p* = 0.036) and old age and very old age were independent risk factors for mortality (odds ratio (OR) and 95% confidence interval (CI) 2.3 (1.2–4.4) and 2.1 (1.2–3.9), respectively). *Enterobacteriaceae* spp. were isolated more frequently in the very old aged patients, but new temperature rise was observed less frequently in this category of patients. Although VAP prevalence is not related to age, ICU admission due to respiratory tract infections are increasing. A French study investigating trends in intensive care admissions for respiratory tract infections in the elderly revealed that ICU hospitalizations for respiratory infections increased 2.7 fold from 2006 to 2015 (*p* = 0.0002) (28). The greatest increases in the use of ICU resources were for the 85–89 and ≥90 years old groups, which corresponded to increases of 3.3 and 5.8 folds, respectively.

### Urinary Tract Infections (UTIs)

While bacteruria is rarely seen in young men, its prevalence reaches ≥5% in those ≥65 years in the community setting. On the other hand, bacteriuria occurs in 5–10% of women aged >65 years as compared to 2–5% of young women (7). In the absence of typical clinical symptoms of an infection, it is important to differentiate asymptomatic bacteriuria from symptomatic bacteriuria.

Elderly patients (>65 years) may have a urinary source of infection in almost 30% of all septic patients in the same age group. Urinary tract infections are the second most common infection that leads hospitalization of elderly after pneumonia (10). In the critically ill elderly, UTI is almost always related with an indwelling urinary catheter and *E. coli* is usually the most common infectious agent followed by *Candida* spp., *Enterococcus* spp., and *P. aeruginosa*. In these groups of patients with fever and/or other signs compatible with infection, urine and blood cultures should always be obtained. However, urinary catheter colonization frequently occurs after hospitalization, thus the urine sample should always be obtained after replacing urinary catheter. Multidrug resistance particularly in the gram-negative bacteria, is challenging and may cause treatment failures. These include extended-spectrum beta-lactamase (ESBL) production and/or carbapenem-resistant Enterobacteriaceae and recently emerging colistin-resistant gram-negative bacilli (29). In an elderly critically ill patient with an indwelling urinary catheter, a persistently sterile urine culture should not be attempted since repetitive courses of antibiotic therapy simply select more resistant bacteria. On the other hand, in a critically ill patient, it proves extremely difficult to differentiate between asymptomatic bacteriuria and real UTI (29). Clinical instability usually would require a course of antimicrobial therapy depending on the susceptibility of the bacteria isolated from urine. In case of candiduria, changing, or removing the urinary catheter is strongly recommended (11). Unless a high risk factor for dissemination is present (e.g., neutropenia, invasive urologic manipulation), antifungal treatment is not justified for candiduria.

### Bacteremia and Sepsis-Related Infections

Bacteremia is often associated with a central line in the hospitalized elderly patients and methicillin-resistant *Staphylococcus aureus* is the most common pathogen in this cohort (12). Sepsis is also more frequently observed in the elderly. A longitudinal observational study including more than 10 million adult sepsis patients over 24 years in 500 US acute care hospitals revealed that while people aged 65 years and older make up about 12% of the American population, they make up 65% of sepsis cases yielding a relative risk of 13.1 (95% CI, 12.6–13.6) compared with younger patients (19). Case-fatality rates increased linearly by age with an average value of 27.7% in the elderly vs. 17.7% in patients younger than 65 years, and age was an independent predictor of mortality [OR 2.26 (95% CI, 2.17–2.36)]. Elderly sepsis patients died earlier during hospitalization and those who survived were less likely to return home (54 vs. 76%). However, they were more likely to be discharged to a non-acute health care facility (37 vs. 15%). In a recent prospective multicenter study with 1,490 elderly patients, the investigators showed that the hospital mortality was significantly higher in very old (>80 years) as compared with those 65–79 years (30). Prompt therapy for sepsis within the first 6 h was associated with reduced mortality in the very old.

In addition, cognitive impairment and functional disability i.e., post-intensive care syndrome (PICS) is very frequent in elderly sepsis survivors with an OR of 3.3 (95% CI, 1.5–7.3). In contrast, non-sepsis general hospitalizations were associated with no change in significant cognitive impairment. This physical and cognitive decline persisted for up to 8 years (20). Sepsis does not only affect the patient, but also the family members or caregivers. It has been reported that especially wives of older sepsis survivors were at greater risk of developing depression (21).

### *Clostridioides* (Formerly Clostridium) *difficile* Infection (CDI)

While the incidence of CD*I* has dramatically increased during the last two decades, the highest mortality and morbidity have been seen in elderly and critically ill patients (13). Over 80% of deaths due to CDI occurs in those >65 years of age (31). Although age is a significant risk factor for CDI, the high burden in elderly has been linked to more frequent interaction with health care systems, frequent exposure to antibiotics, immunosuppression and accompanying co-morbid conditions (22). Recurrences are frequent and may occur in 20–30% of cases. Older patients are more prone to develop recurrence and other risk factors include female sex, previous antibiotics, proton pump inhibitors, or corticosteroids within 90 days preceding the CDI diagnosis (32). A recent analysis indicated that overall health status including infections, health care utilization, acute conditions in the past year, and frailty indicators are the most important determinants of CDI risk in an elderly population. Thus, an elderly person in critical condition will be more likely to have a severe CDI than a person with similar age or older without any comorbidity or previous hospitalization (33).

### Liver Diseases

Human and experimental studies suggest that, in comparison to other organ systems the liver ages fairly well. However, aging is associated with a variety of morphological changes in liver for which the underlying mechanisms are still unclear. The classical appearance of the liver in the elderly is known as “brown atrophy” where the brown color results from the accumulation of highly non-oxidized insoluble proteins known as lipofuscin, which are stored in hepatocytes. The accumulation of these high cross-linked proteins is thought to be associated with chronic oxidative stress and impaired destruction of damaged denatured proteins (34). The decline in mitochondrial capacity with aging has been the underlying cause of many of the changes observed in the function of hepatocytes and macrophages, both contributing to the prevalence and severity of chronic liver diseases. Along with the increase in prevalence of various metabolic diseases, obesity, and cellular senescence in elderly, the liver becomes more susceptible to damage from alcohol, drugs, and toxins. Thus, aging becomes a major risk factor for the development and prognosis of several chronic liver diseases and conditions including non-alcoholic fatty liver disease, alcoholic liver disease, hepatitis C, and for an increased susceptibility to develop fibrosis and cirrhosis (35).

### Other Chronic Diseases Predisposing Infection

The prevalence of increased chronic diseases (e.g., chronic kidney disease, heart failure, lung failure), along with physiological changes caused by aging, frailty, and nutritional problems lead to increased frequency and severity of infections in geriatric patients (8, 36).

The incidence of chronic and end-stage renal disease and the need for dialysis increases with age. Although it remains controversial whether older patients with peritoneal dialysis have increased risk of peritonitis than their younger counterparts, there is general consensus that risk of recurrence of peritonitis and short-term mortality are high in this group of elderly (37).

Heart failure is another chronic disease that increases with age. Elderly patients who are hospitalized for the management of heart failure are more prone to infection due to multiple organ system disorders. Pneumonia, intraabdominal acid-related peritonitis, and dermatitis due to lower extremity edema are among the most common infections in these patients (38).

### Fever of Unknown Origin (FUO)

The etiology and related diagnostic workup of FUO is somewhat different in the elderly patients. Infection may be responsible up to half of the cases including tuberculosis and infective endocarditis (39). Tuberculosis tends to be extrapulmonary in older patients (13). The earlier assumption that malignancies would be more commonly observed in older patients as a cause of FUO has not been confirmed in more recent series (13). One of the important characteristics of patients with FUO eventually identified with an infectious etiology is that these patients tend to be infected with more drug-resistant microorganisms (13). This may be due to accompanying co-morbidities particularly in the critically ill such as those with renal and/or hepatic failure, and previous exposure to antimicrobials. Thus, an aggressive diagnostic approach may be required to identify a potentially life-threatening infection in a severely ill, elderly patient. Such approach would require radiological imaging including CT, MRI and ultrasonography, and biopsy and culture from a potential focus of pathology. However, in early phases of inflammation or infection, the anatomical changes may take time to be visualized. The distinction between active foci of infection and residual changes may not be appropriately distinguished by the aforementioned methods. Thus, a fluorodeoxyglucose-positron emission tomography integrated with computed tomography (FDG-PET/CT) has been recommended to be a part of diagnostic algorithm in patients with FUO when other diagnostic clues are lacking (40).

## Challenges in the assessment of infection in the critically ill elderly patients and triage for ICU admission

Classical symptoms and signs such as fever, cough, dysuria, etc. are not that common in the elderly (7). Despite the fact that fever may be absent or blunted in 20–30% of elderly infected patients, its detection in a geriatric patient should be taken seriously for the evaluation of infection, especially when compared to a younger patient (41). Instead, infections are usually presented with changes in mental status including confusion, obtundation, agitation, delirium, poor oral intake, malaise, falls, urinary incontinence, hypothermia, etc. Presence of hypothermia has been found to be a predictor of mortality in septic elderly patients (42). These atypical findings can also be observed in non-infectious diseases in the elderly making the diagnosis even more difficult. In addition, due to dementia or altered mental status, a detailed history may not usually be obtained, and a thorough physical examination and diagnostic tests are difficult to perform, either. High blood cell count with left shift, acute phase reactants, such as procalcitonin and C-reactive protein have similar diagnostic efficacy in the elderly and the younger patients (7, 43, 44). Thus, they can be considered as adjunctive diagnostic tools for infection, however with limitations. Due to the difficulty in diagnosis, infection could remain unnoticed, therefore the threshold for diagnosis and treatment including ICU admission should be low. The decision whether to accept or decline admission of an elderly patient to the ICU is quite challenging. There is almost no guideline for this specific population and the number of studies is very limited (45). Regardless of the age factor, as with any other medical treatment, the decision to admit a patient to an ICU should be based on the concept of potential benefit. Table 3 summarizes the most important factors to be considered for triage for admitting elderly patients to the ICU (46). A cluster-randomized clinical trial investigating the effect of a protocol for systematic ICU admission in critically ill elderly patients showed no benefit on 6-month mortality compared with usual practice, although a significantly higher ICU admission rate was observed (47). Therefore, further studies and guidelines are needed to clarify admission and discharge criteria for the elderly with or without infection.

**Table 3:** Factors to be considered for triage of admitting elderly patients to the ICU (46).

Diagnosis and severity of infection
Coexisting dieases
Physiological reserve
Expected prognosis of the acute illness and the underlying chronic conditions
Availibility of a suitable treatment
Response to trestment to date
Recent cardiopulonary arrest
Anticipated quality of life
Patient's wishes
Bed availability
Staff workload

## Management of infected critically-ill elderly patient

General principles do not differ in the management of the infected critically ill elderly patients than the younger patients. Sepsis resuscitation and management bundles should be started as early as possible and compliance to the guidelines and bundles have been shown to improve survival over the entire age groups (48). In the recently updated “Surviving Sepsis Campaign” bundle, the most important change is the combination of bundles into a 1-h bundle, i.e., obtaining blood cultures, measuring serum lactate level, administration of antibiotics, 30 mL/kg fluids on average and vasopressors in case of severe hypotension. However, fluid administration beyond initial resuscitation requires careful assessment of the patient in terms of fluid responsiveness, since positive fluid balance is harmful, as well (49).

### Antimicrobial Stewardship

Appropriate antibiotic therapy and effective source control as early as possible are the two vital components of the management of sepsis and serious infections such as VAP. Antibiotic management in the infected critically-ill patient require careful individualized optimization paying attention to various factors including comorbidities, the acute illness, use of continuous renal replacement, and other extracorporeal therapies, changes in drug pharmacokinetics and pharmacodynamics, drug interactions and potential toxicities (14). In addition, food-drug interactions can occur especially during enteral feeding which necessitates suspending feeding for some periods. Therapeutic drug monitoring and close follow up of drug adverse effects are especially important in the elderly (15). However, the principle of aggressive dosing to achieve maximal therapeutic dose should not be sacrificed to avoid potential adverse effects. Pharmacotherapeutic management of the critically ill patient is complex as it covers not only antibiotics, but other drugs commonly used in the ICU as vasopressors, sedo-analgesics, hypnotics, and even prophylactic drugs for venous thromboembolism and stress ulcers.

### Preventive Strategies

Waning immunity in the elderly, not only causes increased prevalence of infections, but may also hamper the vaccine efficacy. However, vaccination against several infections has a critical role in protecting elderly from these infections (50). Recommendations for preventing against *S. pneumoniae* infections vary between countries, but usually include sequential application of 13 valent conjugated pneumococcal vaccine given before 23-valent polysaccharide vaccine (11). Recommended age for these vaccines again differs by countries, but usually is for those >65 years (23). Annual vaccination against influenza with tri- or quadri-valent vaccine is recommended for those >60, however in some European countries (e.g., Austria, Czech Republic, Malta, Slovenia) and in US, the vaccine is available without any age restrictions (>6 months). The controversy about immunization strategies partially reflects the fact that clinical efficacy of influenza vaccine is difficult to determine particularly in the elderly since parameters used in clinical studies are different for each study in each influenza season (50). A meta-analysis indicated that clinical protection does not persist throughout the year after vaccination in elderly (51). Nevertheless, since elderly people are highly vulnerable to complications of severe influenza, they are the main focus of prevention strategies (52). A new universal influenza vaccine (H1ssF_3928) enters human trials and if it proves effective it can provide long-lasting protection (thus no further need for annual vaccination) for all age groups from multiple influenza subtypes (see. https://www.nih.gov/news-events/news-releases/nih-begins-first-human-trial-universal-influenza-vaccine-candidate). A live attenuated zoster vaccine has been available since 2006 and shown to reduce disease incidence by 51.3% and postherpetic neuralgia by 66.5% after single shot application (53). However, the protective efficacy wanes over time and is lower in those >80 years. A new recombinant zoster vaccine has recently been licensed in US and Canada and provides efficacy in all age groups and immunocompromised patients; the latter is a contraindication for live attenuated vaccine (54).

### Mechanical Ventilation

Weaning from mechanical ventilation is difficult in the elderly and increased age is an independent risk for prolonged mechanical ventilation (). Duration of ICU stay and mechanical ventilation was reported to be 5 and 9 days longer, respectively, in patients older than 70 years of age hospitalized due to acute lung injury Older age was also an independent predictor of in hospital mortality with a hazard ratio of 2.5 (95% CI, 2.0–3.2) (55). Aging leads to changes in respiratory physiology as decrease in compliance, diaphragmatic strength, and response of the respiratory centers (5). Underlying COPD, chronic cardiac diseases, presence of sarcopenia and malnutrition contribute to difficulty in weaning, as well. Prolonged mechanical ventilation increases VAP by 1–3% for each extra day of mechanical ventilation (16). Mechanical ventilation is a risk factor for myopathy of critical illness which itself prolongs mechanical ventilation thus creating a vicious circle. Therefore, strategies to reduce duration of mechanical ventilation in critically ill elderly is of utmost importance.

### Bed Sores and Pressure Ulcers

Decreased mobility and bedridden status in critically ill elderly patients predispose an increased incidence of pressure ulcers which are major causes of morbidity and mortality and significant financial burden. The best management approach is to alleviate the risk factors particularly minimizing the prolonged pressure, and keeping the skin clean and dry. However, once a pressure ulcer occurs careful evaluation and staging should be performed. For microbiological documentation, which will be necessary in patients with persistent infection, swab cultures should be avoided and only tissue samples should be cultured. Systemic antibiotic therapy is required for sellulitis and in patients with signs of systemic infections in addition to bed sores. Presence of osteomyelitis would also necessitate parenteral antibiotic therapy along with effective devitalized tissue debridement. Local antiseptics should be applied only in the short term since they may interfere with wound healing. Topical antibiotics have no role for the treatment. Several dressings and topical agents are available and can be effectively used, but none of them have been shown superiority than others. Similarly, negative pressure wound therapy with application of a foam dressing inserted into the wound which is then covered with a film and a vacuum is created by a suction device showed an advantage over the other types of dressings. An extensive review has recently been published about management of pressure ulcers (17).

### Emerging Management Issues for *C. difficile* (CDI) Infection

Despite elderly patients being at a higher risk of developing CDI, the management strategies do not differ between age groups (56). Existing therapeutic approaches include discontinuation of the antibiotics, fluid and electrolyte resuscitation, and therapy with either oral vancomycin or fidaxomycin. Oral metronidazol can be an alternative in mild to moderate cases. A human monoclonal antibody against *C. difficile* toxin B, bezlotoxumab, has been approved for preventing CDI recurrences in high risk patients. In a placebo controlled trial, bezlotoxumab decreased CDI recurrence by 51% than placebo in patients >65 years. Heart failure was significantly higher in the bezlotoxumab group and would require particular attention in the elderly (57). Fecal microbiota transplantation (FMT) is recommended for patients with multiple recurrences who fail the appropriate antibiotic therapy (58). In a recent small, single center, randomized trial, FMT after 4–10 days vancomycin treatment was found to be superior than either vancomycin or fidaxomycin therapy in patients with recurrent CDI (59). Despite the modest sample size of the study, these results may underly the importance of this practice over anti-Clostrioides antibiotics. Probiotics, despite several studies of low-to-moderate quality and meta-analyses available, cannot be recommended of preventing CDI (56, 58).

### Delirium and Altered Mental Status

Elderly critically ill patients are at high risk for delirium, which has an average frequency of 60% and increases length of stay and mortality (60). Drug-induced delirium is well-described with sedatives, anticholinergics and narcotics but can also occur, albeit less frequently, with various antibiotics including cephalosporins, macrolides, and rarely with quinolones (61, 62). The effect of antibiotics on central nervous system functions should be meticulously evaluated in a critically ill patient with altered mental status (63).

### Quinolone Use and Risk for Aortic Aneurism and Dissection

A recent warning by FDA noted that observational studies suggested an association between quinolone antibiotic use and aortic aneurysm and dissection (64). Particular risk groups included those patients with hypertension, peripheral atherosclerotic disease and advanced age among others. Although the casual role of quinolones for this complications is not clear, it may be wise to withhold quinolones in the elderly if any other antibiotic options are available.

## Conclusions

Managing infections in severely ill, elderly patients is a very complex issue which requires teamwork. A prompt, intensive and laborious diagnostic workup is essential, and an empirical, antimicrobial regimen may need to be applied as early as possible, covering presumptive offending pathogens in order to prevent mortality. Once a microbiological diagnosis is made, the initial therapy may switch to be targeted and streamlined. Since several non-infectious complications in older patients may alter the course and severity of infections in the elderly, a comprehensive management strategy would be unavoidable.

## Author Contributions

All authors listed have made a substantial, direct and intellectual contribution to the work, and approved it for publication.

### Conflict of Interest Statement

The authors declare that the research was conducted in the absence of any commercial or financial relationships that could be construed as a potential conflict of interest.
